# Hybrid video-assisted thoracoscopic radiofrequency ablation of recurrent ventricular tachycardia in a patient with desmoplakin cardiomyopathy

**DOI:** 10.1016/j.hrcr.2024.04.005

**Published:** 2024-04-17

**Authors:** Stephan A.C. Schoonvelde, Frank R.N. van Schaagen, Alexander Hirsch, Michelle Michels, Sing-Chien Yap

**Affiliations:** ∗ Department of Cardiology, Thorax Center, Cardiovascular Institute, Erasmus MC, Rotterdam, The Netherlands; † Department of Cardiothoracic Surgery, Thorax Center, Cardiovascular Institute, Erasmus MC, Rotterdam, The Netherlands; ‡ Department of Radiology and Nuclear Medicine, Erasmus MC, University Medical Center Rotterdam, Rotterdam, The Netherlands

**Keywords:** Ablation, Arrhythmogenic cardiomyopathy, Desmoplakin cardiomyopathy, Video-assisted thoracoscopy, Ventricular tachycardia

**Key Teaching Points**

• Drug-refractory ventricular arrhythmias are a challenge in patients with desmoplakin (DSP) cardiomyopathy.
• In patients with DSP cardiomyopathy, there can be an extensive epicardial substrate that forms the basis of reentrant ventricular tachycardia.
• When percutaneous epicardial access fails owing to pericardial adhesions, a minimally invasive hybrid video-assisted thoracoscopic ablation is a suitable alternative.

## Introduction

Desmoplakin (DSP) cardiomyopathy is an inherited left-dominant arrhythmogenic cardiomyopathy associated with heart failure, epicardial ventricular fibrosis, and ventricular arrhythmias (VA). Inflammation may play a potential role in its pathophysiology given that myocarditis-like episodes (“hot phase”) occur, particularly in women, which are characterized by chest pain episodes accompanied by electrocardiogram (ECG) changes and cardiac troponin release.^1^ Because patients with DSP cardiomyopathy are at high risk of sudden cardiac death owing to VA, the current guidelines recommend an implantable cardioverter-defibrillator (ICD) in patients with late gadolinium enhancement on cardiovascular magnetic resonance (CMR) and/or left ventricular ejection fraction <45%.^2^ Despite antiarrhythmic therapy, patients with DSP cardiomyopathy may present with recurrent episodes of VA and ICD therapy. Epicardial ventricular tachycardia (VT) ablation plays an important role in the invasive management of recurrent VA and has been associated with good clinical outcomes.^3^, ^4^, ^5^ However, the experience with epicardial VT ablation in DSP cardiomyopathy patients is limited in the current literature. We present a case of video-assisted thoracoscopic radiofrequency epicardial VT ablation in a patient with DSP cardiomyopathy with recurrent drug-refractory monomorphic VT.

## Case report

**Figure 1:**
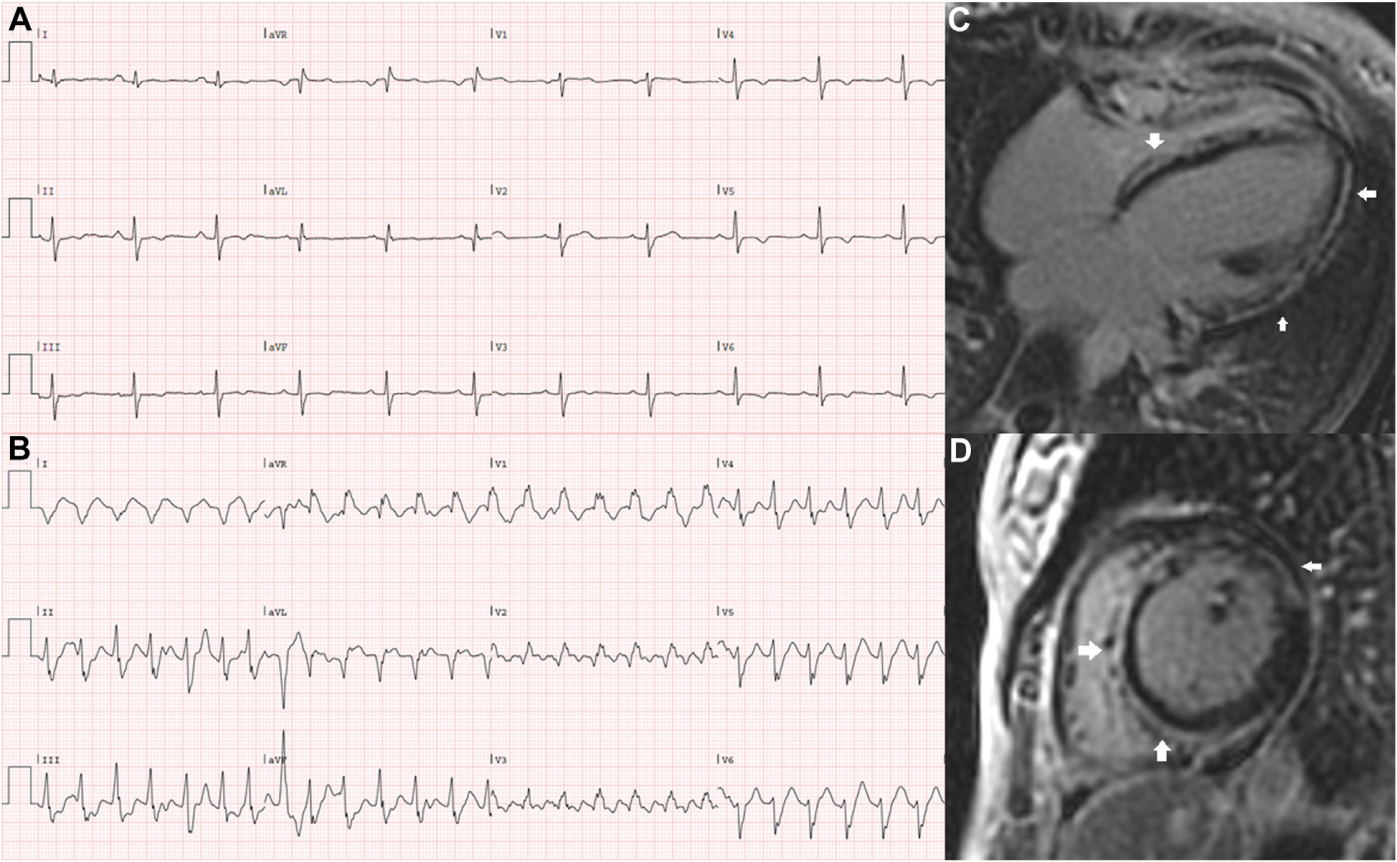
**A:** Resting electrocardiogram (ECG) showing a sinus rhythm with a prolonged PR interval (206 ms), minor intraventricular conduction delay (QRS 121 ms), and diffuse inferolateral repolarization abnormalities. **B:** ECG showing an episode of monomorphic ventricular tachycardia of 157 beats per minute originating from the anterolateral left ventricle with fusion beats. **C, D:** Extensive ring-like late gadolinium enhancement (*arrows*) of the left ventricle depicted in the 4-chamber (**C**) and short-axis (**D**) view.

We present a 32-year-old woman who was diagnosed with mild dilated cardiomyopathy at the age of 16 during cardiac screening for recurrent syncope. Her monozygous twin sister had a similar phenotype. Her first ECG showed nonspecific repolarization abnormalities. Holter monitoring demonstrated single ventricular ectopic beats and nonsustained VT. CMR (performed at 17 years) showed already extensive epicardial “ring-like” late gadolinium enhancement of the left ventricle (LV) (Figure 1C and 1D). She was treated with bisoprolol and perindopril. Targeted next-generation sequencing of cardiomyopathy-related genes performed when the patient was 22 years old revealed a heterozygous c.4608_4612del5, p.(Arg1537fs) variant in the *DSP* gene (NM_004415.2), encoding the desmosomal protein desmoplakin. During follow-up her left ventricular ejection fraction progressively declined to 30% and at the age of 22 years a prophylactic dual-chamber ICD was implanted. After ICD implantation, she experienced multiple episodes of appropriate ICD therapy owing to sustained VT and ventricular fibrillation. Consequently, her beta-blocker dose was increased. Her recent ECG demonstrated mild conduction disorders and inferolateral repolarization abnormalities (Figure 1A). Recently she experienced another ICD shock and presented at the emergency room with a sustained VT (157 beats/min) episode, which was terminated with intravenous amiodarone (Figure 1B). The VT exit site seemed to be localized to the mid lateral LV based on her 12-lead ECG. Her medication was switched to sotalol, and later to amiodarone. One month later she experienced an ICD storm, and she was planned for an endo-epicardial VT ablation owing to the high likelihood of an epicardial substrate in DSP patients, as seen on the patient’s CMR with extensive epicardial late gadolinium enhancement.

**Figure 2:**
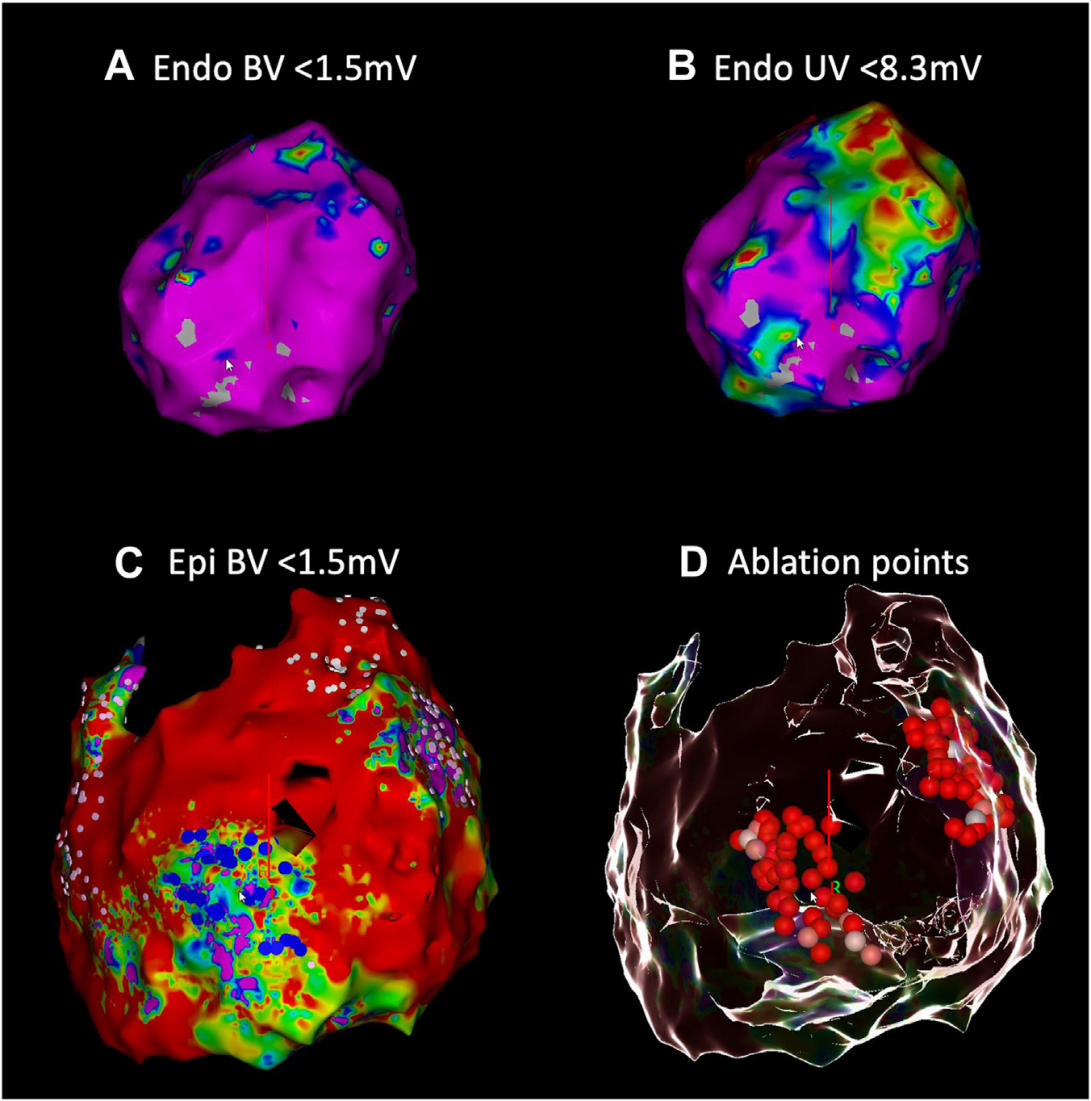
**A:** Left lateral view of the endocardial bipolar voltage map showing no significant low-voltage areas (<1.5 mV). **B:** Same map as in (A) demonstrating extensive areas with <8.3 mV unipolar voltage, suggesting a large epicardial area with low voltage, predominantly within the mid to basal anterolateral left ventricle. **C:** Epicardial bipolar voltage map demonstrating extensive pathologic substrate with scar tissue (*red*) and low-voltage areas with late potentials (*dark blue dots*) and fractionations (*pink dots*). Some parts of the anterior left ventricle could not be reached owing to adhesions. **D:** Glass mode view demonstrating the location of ablation points with a similar view as in C. The 2 areas with low voltages were targeted. BV = bipolar voltage; UV = unipolar voltage.

The procedure was performed during general anesthesia. Unfortunately, we failed to acquire subxiphoid percutaneous epicardial access using the Sosa technique despite multiple attempts. Contrast injection did confirm proper position of the Tuohy needle in the pericardial space by showing a thin outline of the pericardium, but the guidewire could not be advanced, potentially owing to pericardial adhesions. We decided to perform a detailed endocardial LV voltage map (CARTO 3; Biosense Webster, Irvine, CA) during sinus rhythm, which demonstrated no low bipolar voltage areas (<0.5 mV) (Figure 2A); however, the unipolar voltage map suggested a large epicardial low-voltage area in the basal anterolateral region (Figure 2B). Furthermore, the clinical monomorphic VT (cycle length 460 ms) was inducible with programmed ventricular stimulation. The earliest activation of the VT was at the basal lateral LV. It was concluded that the substrate was located at the epicardial site, and no attempt to ablate was performed endocardially. She was rescheduled for a hybrid video-assisted thoracoscopic ablation.

**Figure 3:**
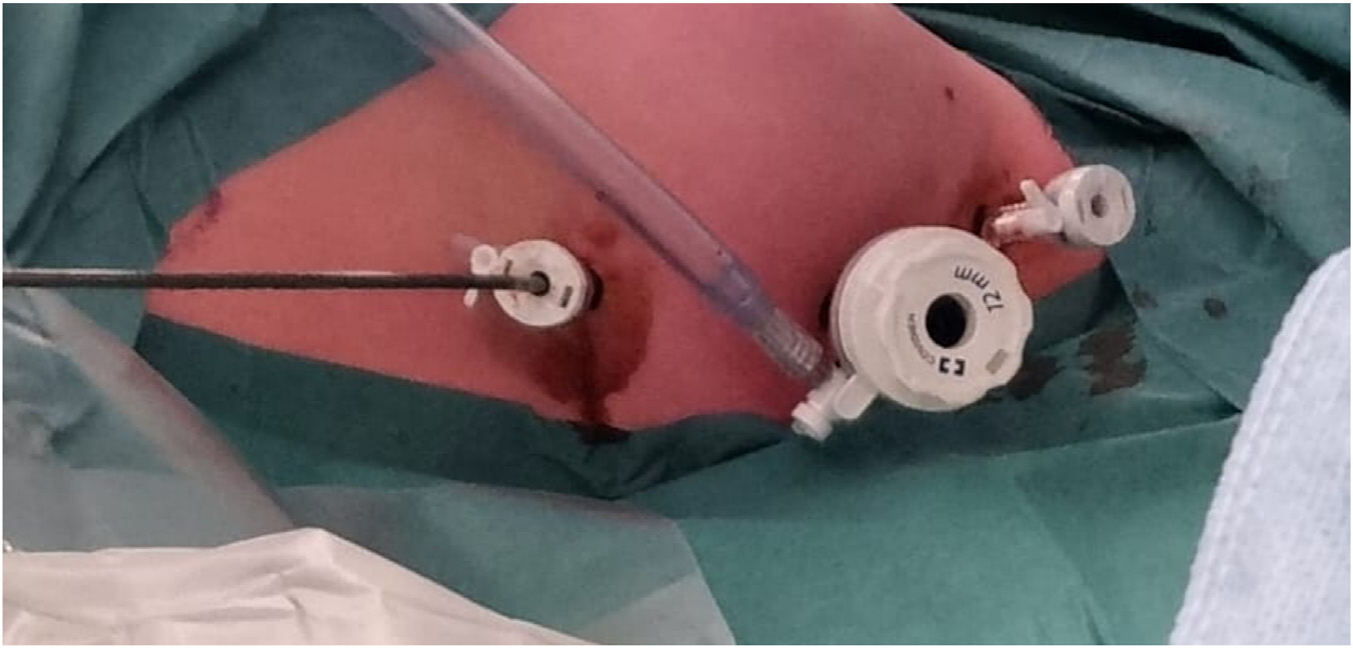
Left-sided thoracoscopy. The camera port is inserted in the fourth intercostal space, the working ports are installed in the third and sixth intercostal spaces.

During the hybrid video-assisted thoracoscopic ablation procedure, surgical access to the pericardial space was achieved via a left-sided thoracoscopic approach by the cardiothoracic surgeon (Figure 3). A 5-mm camera port and 2 5-mm working ports were installed in intercostal spaces III, IV, and VI. The pericardium was opened anteriorly to the left phrenic nerve at the lateral side of the LV. A guidewire was placed in the pericardial space via the lower thoracoscopic port under direct visualization. An Agilis steerable sheath (St. Jude Medical, Plymouth, MN) was placed in the pericardial space over the guidewire. A decapolar catheter was placed into the coronary sinus and a quadripolar catheter in the right ventricle. A high-density electroanatomical contact map was created using an Octaray (Biosense Webster) catheter and CARTO 3 system (Biosense Webster). We specifically did not use an impedance-based mapping system, as this requires a navigation patch on the left mid-axillary line to ensure proper 3D tracking (which was not possible with the ports). The mapping was at times hampered by difficulties with collecting anatomy points, most likely owing to magnetic distortion or absence of conductive liquid around the splines.^6^ By slowly mapping, this issue was overcome. Finally, the epicardial voltage map demonstrated extensive pathologic regions of scar and low-voltage areas (Figure 2C). Parts of the pericardial space could not be reached owing to adhesions (Figure 2C). The anterior LV showed a low-voltage area with late potentials (dark blue dots), and the basal anterolateral LV had a low-voltage area with extensive fractionation (pink dots). We performed extensive epicardial radiofrequency ablation to eliminate all abnormal potentials in these 2 areas (Figure 2D). By homogenization of these areas, we eliminated potential channels of a reentrant circuit. Temperature- and flow-controlled radiofrequency ablation was performed with a QDOT Micro ablation catheter (Biosense Webster) using the following settings: QMODE, 50 W, target temperature 47°C, flow rate 4–15 mL/min (depending on interface temperature). At the end of the procedure, no VT could be induced with programmed ventricular stimulation (2 drivetrains, 2 extrastimuli). The ventricular effective refractory period was 290 ms (under amiodarone). The postoperative period was uncomplicated and after 5 days she could be discharged to home. Four months after the procedure, she experienced pericarditis, for which she received colchicine and intermittent nonsteroidal anti-inflammatory drugs, with good effect. There was no recurrence of sustained VT during a follow-up of 9 months while continuing to use amiodarone and bisoprolol.

## Discussion

The management of VT in patients with DSP cardiomyopathy is challenging owing to its high arrhythmogenicity, which may persist despite antiarrhythmic therapy. The patient in this case presented with recurrent episodes of VT despite the use of maximally tolerated beta-blockade, amiodarone, or sotalol. Given the increasingly frequent appropriate ICD therapy in the patient of our case, alternative management must therefore be considered.

Endo-epicardial ablation is the cornerstone of invasive management in nonischemic cardiomyopathy patients with VA, but there is limited experience in patients with DSP cardiomyopathies.^3^, ^4^, ^5^ VT ablation in DSP cardiomyopathies is sparsely described in literature, and this is the first report of a hybrid ablation approach applied to a patient with DSP cardiomyopathy. When assessing how to best treat an individual patient, it is important to understand the specific etiology of the cardiomyopathy, as distinct pathophysiologies may lead to different patterns of fibrosis and electrophysiological scar characteristics.^5^ Therefore, a patient-tailored understanding and targeted approach is crucial in deciding the optimal treatment strategy for each patient.

The discordance between the endocardial and the epicardial substrates in DSP cardiomyopathy is remarkable. Epicardial fibrosis is common in DSP cardiomyopathies and may be extensive, as shown in our patient.^1^^,^^7^ Therefore, epicardial ablations may be required owing to the high VA burden in DSP cardiomyopathies. However, the association with inflammatory episodes, which seems to be specific to patients with DSP cardiomyopathy (the so-called “hot phase”),^1^^,^^8^ might lead to the development of pericardial adhesions, which may form a technical challenge for pericardial access in DSP patients. The patient in this case did not have any prior cardiac surgery, and it is, therefore, possible that the pathophysiology of these pericardial adhesions may be the result of myocardial inflammation. One recent study has also reported pericardial adhesions in a patient with DSP cardiomyopathy requiring epicardial surgical access.^4^

Hybrid video-assisted thoracoscopic ablation is an alternative when percutaneous epicardial access is hampered and is an effective treatment in arrhythmogenic cardiomyopathies.^5^^,^^9^ This technique allows for a safe and effective approach in arrhythmogenic cardiomyopathies, particularly in the presence of pericardial adhesions that may make other approaches challenging.^10^^,^^11^ Furthermore, a surgical approach has not been associated with worse outcomes compared with percutaneous access.^12^ Just like in our case, this technique is often applied after other prior approaches have been attempted. Our patient responded well to the procedure, having complete and persisting resolution of sustained episodes of VA as assessed from her ICD, which is contrasted by the high VA burden before the procedure.

When performing hybrid video-assisted thoracoscopic ablation, the operator must be aware that the commercially available electroanatomic mapping systems have their specific pitfalls regarding the epicardium.^6^ Systems using impedance mode to track catheters may encounter difficulties owing to single-lung ventilation and high CO_2_ pressure in the thorax, which affects thoracic impedance. The CARTO 3 system, which is one of the most used electroanatomic mapping systems, uses hybrid current-based and magnetic information for catheter tracking and map visualization. The location of the catheter is determined by 3 magnetic fields. Current-based information renders catheter localization more precise. There have been reports that operators were unable to use high-density mapping catheters such as Octaray for the collection of anatomy points during epicardial access.^6^ This may be owing to magnetic distortion and/or the lack of conductive fluid around splines. In our case, we experienced difficulties in the acquisition of the map, but by slow movement during mapping, we were able to construct a high-density map.

Pericarditis after surgery involving the pericardium, which occurred in our patient, has infrequently been reported.^13^^,^^14^ Its onset can be months after the surgical procedure. One review described an incidence of 3% after hybrid ablation despite prophylactic use of colchicine.^15^ Overall, its incidence seems to be low after a hybrid approach. Our patient responded to the standard treatment for pericarditis, with flare-ups occurring possibly owing to initial underdosing of colchicine (0.5 mg once daily instead of twice daily with patient weight of 71 kg) because of gastrointestinal side-effects. Later increased dosing resulted in persisting resolution of symptoms.

## Conclusion

To the best of our knowledge, this is the first case of hybrid video-assisted thoracoscopic epicardial ablation to treat drug-refractory recurrent VT in a patient with DSP cardiomyopathy in whom percutaneous epicardial access had failed. This case demonstrates that a hybrid approach is feasible and a safe alternative for patients with an epicardial substrate.

## Disclosures

SCY has received honoraria from Boston Scientific, Medtronic, Abbott, Biotronik, Acutus Medical, and Sanofi. In addition, he has received institutional research grants from Medtronic, Biotronik, and Boston Scientific. AH received an institutional research grant and consultancy fees from GE Healthcare and speaker fees from GE Healthcare and Bayer. He is also a member of the medical advisory board of Medis Medical Imaging Systems and was MRI corelab supervisor of Cardialysis BV until 2022. MM received a research grant and speakers fee from Bristol Myers Squibb, consultancy fees from Cytokinetics, and speakers fee from Pfizer. The other authors declare that they have no competing conflicts of interest.
